# Using Rhythmic Notation and Musical Analysis on Animal Communication: A Case Study on Sperm Whales

**DOI:** 10.1111/nyas.70210

**Published:** 2026-01-26

**Authors:** Mia Davitt, Macrae Eckelberry, Max Davitt, Lara S. Burchardt

**Affiliations:** ^1^ Independent Scholar Los Angeles California USA; ^2^ Institut Für Theoretische Biologie Humboldt‐Universität zu Berlin Berlin Germany

**Keywords:** musicality, rhythm, rhythmic notation, sperm whale, vocalization

## Abstract

Western music notation, a language of symbols representing various parameters in music, can be used to describe and analyze existing musical performances. Rhythmic elements such as periodicity and categorical rhythm have been studied in sperm whale (*Physeter macrocephalus*) codas, which are short click sequences produced in social interaction. As a case study in the applicability of music notation for animal communication, we transcribed human music, randomly generated rhythms, and sperm whale codas in Western music notation. Music notation categorizes sound elements into a metric hierarchy based on the perception of an isochronous beat in nonisochronous rhythms, a difficult comparison when we cannot know the rhythm perception of nonhuman animals. In accuracy and complexity, the transcriptions of codas showed similar statistics to the human rhythm samples. We demonstrated two modes of musical analysis on the transcriptions of sperm whale codas: tempo variation and motivic variation, and explored how they could be applied in ways that mitigate the subjective nature of interpreting beats. Our sample size was small, and our tools were time‐consuming, so a streamlined approach is needed to fully test the applicability of these tools on a large scale.

## Graphical abstract

Western music notation is a language of symbols which efficiently communicates common elements in music, some of which have been studied in sperm whale (*Physeter macrocephalus*) communication. As a case study in using rhythmic notation on animal vocalizations, we transcribed human music, randomly generated rhythms, and sperm whale codas in Western music notation. This required interpreting a beat in each sample and testing the accuracy of our interpretation.

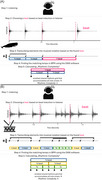

## 1 Introduction

*Western music notation* is a language of symbols that represent pitch, rhythm, and other parameters in music. It was initially developed to be read by performing musicians as an efficient alternative to learning music by ear [1]. The modern‐day standard defaults to a series of rhythmic divisions, organizing time into (from large to small) *measures*, *beats*, and *subdivisions* (glossary of italicized terms is provided in Table 1).

**TABLE 1:** Glossary of terms in both animal communication and Western music notation.

*Term*	*Discipline*	*Definition*
Western music notation	Music	A practice that presents performed music in the form of symbols
Onset	Communication	The beginning of a sound element
Note	Music	A classification of a symbol in music notation representing a sound, its beginning, and its length of sustain (if any)
Inter‐onset interval	Communication	The time span between the onsets of two sound elements; called inter‐click interval in the context of sperm whale vocalizations
Rhythmic value	Music	The sustain of a note expressed as a proportion of the metric beat, represented in music notation by the symbols shown in Figure 1
Rest	Music	A symbol representing the rhythmic value of a space between notes
Staccato	Music	A symbol added to a note to specify a short sound; when a note is staccato, the note's length refers not to sustain but amount of time after the sound
Measure	Music	A short section of music, made up of a specified integer of beats; delineated in music notation by vertical lines dividing groups of notes
Beat	Interdisciplinary	Or “metric beat,” an isochronous pulse perceived in music by humans
Isochrony	Communication	A rhythm pattern where inter‐onset intervals are identical between sound elements
Tempo	Interdisciplinary	Frequency of the metric beat, given in Hertz (Hz) (beats per second); beats per minute (BPM) in music notation
Subdivision	Music	Division of a beat into smaller units, most often evenly into two or three parts all equal in time
Beat induction	Communication	The common human process of identifying an isochronous pulse within a rhythm, regardless of whether that rhythm is isochronous or heterochronous; often occurs instinctively
Metric hierarchy	Interdisciplinary	A feature present in many kinds of human music, in which all events are organized around an isochronous beat, or multiples and divisions of the beat
Syncopation	Music	The presence of notes that do not coincide with the metric beat—all deviations from isochrony in human music can be classified as syncopation, but there are varying degrees of complexity
Categorical rhythm	Communication	A rhythm in which durations between sound elements are distributed categorically; explored by focusing on ratio relationships between inter‐onset intervals; typical ratios between sound elements in music: 1:2, 1:3, or 2:3
Motive	Music	A short series of notes that is reiterated and altered to create new musical material in a process called motivic variation

Despite its initial use in performance, music notation has also been used to analyze existing music performances [2, 3, 4]. Among its strengths are widespread literacy in the language, as well as clarity in communicating musical elements that are often of interest.

Several musical elements have been attributed to sperm whale *(Physeter macrocephalus)* codas: short, repetitive patterns of clicks used frequently in social situations [5]. Indeed, the name “coda” was given as a comparison to musical codas, due to the belief that both phenomena came at the end of a longer series of events [6]. This particular attribution is no longer accepted, but concepts like tempo variation [7], categorical rhythm [8], and periodicity [9] have also been studied in codas—all of which have been used to analyze human music as well [3, 10, 11].

As musicians, we visualized these comparisons in the language of music notation, which led to a question: would it be possible to transcribe sperm whale codas in this language? If so, it would be necessary to interpret the rhythms through the lens of meter.

In human rhythm, there is often the perception of an underlying *isochronous beat* [3, 12, 13]. *Isochrony* is the regular occurrence of events; when music is played to a metronome, the isochronous beat is made clear in the regular click of the metronome. However, even without a metronome, humans often tap along to music at a regular frequency [13]. This can be attributed to a process known as *beat induction*, in which a listener recognizes an isochronous beat in a piece of music, regardless of which sounds in the piece coincide with an instance of that beat [3]. The sounds that do not coincide with the beat coincide instead with small integer multiples of the beat. Beyond beat induction, the inference of meter occurs when a listener groups beats together, identifying a stronger downbeat [2]. This musical relationship with time, that is, all notes occurring in relation to a structure based on the perceived metric beat, makes up a larger framework known as *metric hierarchy* [12].

Any document written in Western music notation will communicate metric hierarchy—one cannot easily avoid, for instance, the division of a piece into measures, measures into beats, and beats into subdivisions. The notation of any series of events, then, requires the person transcribing to perform beat induction and contextualize each event within a perceived metric hierarchy.

Can Western music notation, a language that presents all events within a metric hierarchy, accurately describe sperm whale codas? What can we learn about codas based on the answer to the previous question? And finally, what kinds of analysis become available to us when coda sets are transcribed in this way?

We transcribed sperm whale codas, human drum recordings, and randomly generated rhythms in Western rhythmic notation. We measured the accuracy of all transcriptions and analyzed the resulting notations. This is a pilot study, meant to demonstrate methods and the viability of the concept rather than provide statistically significant results of our analyses. We must be cautious here; human perception undergirds the study of beat and metric hierarchy in human music. Such an understanding of nonhuman animal cognition is not available to us yet. While we test the usefulness of constructs such as meter, we will divorce from the results any claims about animal perception of meter in the samples we study.

With that in mind, notations of animal vocalizations could still be quite useful. When studying sperm whale codas, *tempo* (frequency of beats, measured in Hz or BPM) is currently necessarily calculated by comparing the lengths of codas of the same rhythm type [7, 14]. Codas may be of the same rhythm type when their sequences of *inter‐click intervals* (ICIs) do not exactly match, but are proportionally similar—a coda with the ICIs 1s / 1s / 2s would be the same rhythm type as one with the ICIs 2s / 2s / 4s. These rhythm types are given names such as 3+1, the symbol + denoting a pause that exceeds the other ICIs in the coda [5]—not to be confused with the unfortunately visually similar notation for ratios between *inter‐onset‐intervals* (IOIs), such as 3:1 [10]. When a coda with the same ICI proportions is longer, its tempo is considered lower. The resulting measurement of tempo is given in seconds. If many instances of the same coda type were instead transcribed (and the interpretation of “beat” were the same across all transcriptions), tempo could be measured as the frequency of the metric beat. Analyses could be much more granular—one could measure tempo fluctuation within codas, codas of similar but nonidentical rhythm types could be compared, and so on. The interpretation of beat must be consistent across all samples, with the understanding that such an interpretation is subjective, and not endemic to the vocalizations themselves.

We analyzed our results through the lenses of tempo variation and *motivic variation*, with the goal of exploring possible applications of music notation when studying animal vocalizations. There are a few considerations specific to the transcription of sperm whale codas that require further explanation.

**FIGURE 1:**
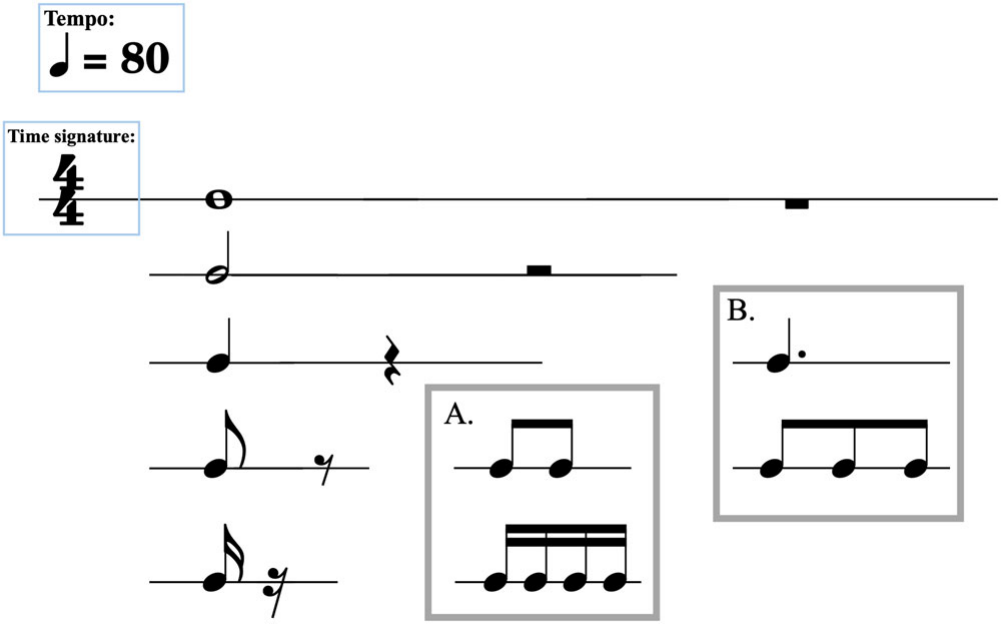
Symbols in Western music notation, organized by rhythmic value. All symbols in a row are the same value. The first symbol in a row represents a note, and the second represents a rest, or pause. Symbols in each row are half the value of the row above it, and twice the value of the row below. The tempo marker indicates which symbol represents the beat, and the frequency of that beat in beats per minute. Time signature refers to the number of beats in a measure. Only the top number is relevant in most cases. The bottom number is useful in the rare case that the tempo and time signature quantify different rhythmic values as the metric beat. Box A: Symbols of the same value, when in succession, are beamed together. Box B: Adding a dot next to any symbol indicates 1½ times the value of the standard symbol.

Note length refers to the rhythmic value of a note and is represented by the symbols shown in Figure 1. Those symbols are named based on their relation to the measure, but the measure's length is defined in relation to note values [1]. To deemphasize this recursive definition, we will refer to a rhythmic value only by its symbol or the multiple of the metric beat it represents. With a sound generator like the human voice, which is capable of sustaining for periods of time, note length instructs the performer how long to hold a note. In the case of *staccato* (a symbol demarcating short notes), the value of the symbol representing a note corresponds not to the length of the sound but to the length of the pause after the sound. All audio transcribed in our research was made up of short sounds and can be thought of as staccato notes. Thus, the distinction between note value and rest length is unnecessary, and we will only refer to the space between sound elements as IOI or, in the case of sperm whales, ICI.

Beat induction occurs instinctively in human listeners, and the results often receive no scrutiny, but in this case, conscious consideration is warranted [3]. When a rhythm has multiple IOIs, how does one identify which is the guiding beat? The answer is heavily dependent on context: rhythm of preceding and following measures, longest IOI, most commonly occurring IOI, accented notes, and so on. However, any integer multiple of the smallest IOI could be counted as a beat without altering the informational content of a transcription.

Any polyrhythm (a rhythm where multiple integer subdivisions of the same span of time are overlaid) can be taken as an example. When listening to a 3 by 4 polyrhythm, in which two isochronous pulses are played at a frequency (frequency of onset, rather than pitch or timbre) ratio of 3:4 with one another, either pulse may be interpreted as the beat [15]. In fact, with careful listening, one can switch back and forth, hearing either pulse as the guiding beat at will.

In human music, there may be a correct—or at least commonly agreed on—way to interpret a piece of music. In animal vocalizations, there can be no such thing. In the case that beat induction in the transcriber did not yield an obvious interpretation, we chose an interpretation willingly, making sure all other instances of the rhythm type in question were interpreted similarly.

## 2 Materials and Methods

### 2.1 Datasets

We transcribed data from three sources: humans, sperm whales, and random generation. The transcriptions are similar to standard sheet music in format and philosophy, with the exception of continuity. We focused on one measure of music at a time, noting the time code of each measure's beginning even when (in the case of human music) the measures succeeded each other in rhythm. Sperm whale codas, unlike most measures in the human rhythm recordings, do not immediately follow one another in time; this was the motivator behind our decision.

Since the methods were novel and time‐consuming, the sample size was necessarily small. Multiple samples with complicated rhythms were selected with the aim of testing transcription accuracy in extreme circumstances. Sampling of sperm whales, especially, was only broad enough to test music notation on a few cases—the goal was to determine if transcribing the rhythms was possible before a larger‐scale implementation of the methods below.

Sperm whale hydrophone recordings were sourced from Watkins Marine Mammal Sound Database, via Woods Hole Oceanographic Institute and the New Bedford Whaling Museum [16]. Human recordings were sourced from Smithsonian Folkways and percussion practice tapes uploaded to YouTube. The collection of human recordings was restricted by the need for linear, single‐drum performances, which were transcribable by the exact same means as those with which we transcribed sperm whale codas. The selection consisted of various American pieces on a single snare drum, and a West African piece on the talking drum [17, 18, 19]. From video confirmation of the snare pieces and the nature of the talking drum piece as a field recording, we confirmed the recordings were only edited at their beginnings and ends. We generated random rhythms by randomly assigning rhythm onsets to a minute‐long Musical Instrument Digital Interface (MIDI) file. Three random recordings were made, each with a different average onset frequency between 0.7 and 2.9 Hz—a lower range than that of codas [9], but one that allowed the chaotic‐by‐nature rhythms to be more easily parsed.

### 2.2 Transcription

**FIGURE 2:**
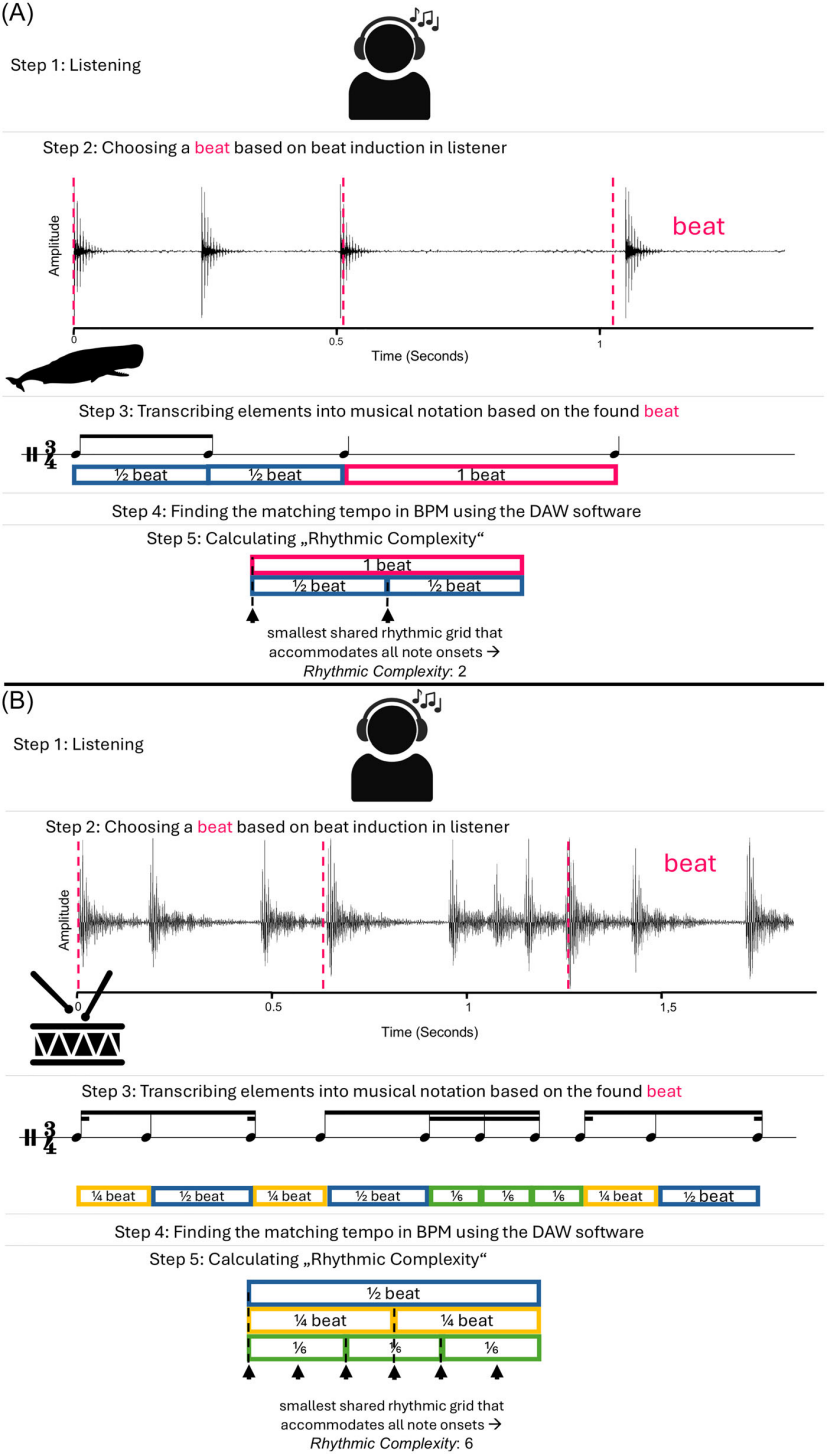
Two examples of the transcription process. Step 1: Listening to the sample. Step 2: Waveform of a sample, a pink dotted line marking the beat as interpreted by the transcriber. Step 3: The transcription in Western music notation. Time signature chosen after beat induction to demonstrate the number of beats in the measure. Below the transcription, each note's value as a proportion of the found beat. Step 4: Finding the correct tempo. Step 5: Rhythmic complexity calculation. All IOIs in the transcribed measure, organized in order of rhythmic value. Black arrows represent the calculation of rhythmic complexity: subdividing the longest inter‐onset interval (IOI) in a way that coincides with all other subdivisions. The number of arrows represents the rhythmic complexity. (A) Waveform from a sperm whale, and its transcription with an average offset of 7 ms. A rhythmic complexity of 2. (B) A waveform of a human playing the snare drum, with an average offset of 19 ms. A rhythmic complexity of 6. Abbreviation: BPM, beats per minute.

For the purposes of transcription, every audio file was transferred into Logic Pro X or Ableton Live, two digital audio workstations (DAWs), each with a useful tempo interface. Sample rate and bit depth of the DAW session matched that of each audio file. Transcriptions were made in MuseScore 4, one measure of rhythm at a time. Figure 2 illustrates the transcription process.

Step 1: We listened to the sample. In audio recordings with various sound sources, we manually identified sources based on timbre, amplitude, and the distinctive reflection pattern of sperm whale clicks [20, 21]. We recorded the time code, in milliseconds, of the first note in each measure.

Step 2: We interpreted a beat in the sample. This generally occurred without issue upon first listening to a measure; factors such as most commonly occurring IOI usually made the interpretation easy. In instances where a beat was not immediately discernible, we visually examined the waveform and experimented with different interpretations. In such cases, a conscious choice was made to interpret the beat in a certain way, and care was taken that all other repetitions of that rhythm would be transcribed in the same manner.

Step 3: We notated the rhythm. After a beat was chosen, the number of beats in the selection dictated the measure length. This was represented by a time signature, where the top number represents the number of beats, and the bottom number identifies which symbol is equal to the value of the beat. In all cases except 2 out of 97, the metric beat was transcribed as the symbol ♩. The two exceptions, both from the recording of a human playing the talking drum, identified the beat as ♪ because the emphasized notes within the measure would not have been accurately demonstrated otherwise.

All sounds that did not coincide with the beat were notated as subdivisions. This also required interpretation in deciding which subdivision of the beat best described the sound in question. To avoid overwrought transcriptions, onsets were generally notated at the maximum resolution of 1/fourth of the metric beat. Subdivisions higher than 4 were only chosen if one or more of these situations arose: there were subdivisions of subdivisions, there were more than four notes within a beat, or an IOI was much shorter than 1/fourth of the beat. In such cases, the lowest subdivision that could describe the note was still selected. The shortest note transcribed was 𝅘𝅥𝅰.

After all recordings were transcribed, we flagged phrases of 3 or more notes that repeated elsewhere in the same species’ dataset, marking them with standardized names that reflected their rhythms.

Step 4: We used the tempo interface of the DAW to select a tempo, making adjustments until the metronome clicks most closely aligned with all sounds that coincided with the found beat, to the precision of 0.5 BPM.

### 2.3 Rhythmic Complexity

Step 5: Also notated was the rhythmic complexity, a measurement we created to fit the needs of this project. The goal of the complexity number was to quickly demonstrate the variety of subdivisions within one measure of a transcription—as opposed to other known systems of quantifying complexity, which focus instead on syncopation or heterogeneity across an entire piece [4]. We here define the complexity number, a positive integer value representing the lowest integer subdivision of the longest IOI in a measure, that could be used to describe every note within that measure.

In determining complexity, we used the longest IOI in a measure as a yardstick, since the length of the measure in beats was susceptible to bias in the transcriber toward human tendencies in rhythmic notation. Rhythmic complexity is calculated independently from time signature, which would leave the result subject to interpretation; rather, it relies only on the note values in the transcription.

The methods are explained visually in Figure 2, but another explanation is this: each note value in a measure was expressed as a fraction of the metric beat. All values were multiplied by the inverse of the largest value (longest IOI). The lowest common denominator of all resulting values was the rhythmic complexity which is the smallest shared rhythmic grid that accommodates all note onsets.

A rhythmic complexity of 1 indicates isochrony. A value of 2 indicates two categories of IOI with a 2:1 relationship with each other (Figure 2A). Rhythms with multiple subdivisions that are indivisible by one another, like Figure 2’s second example, yield a higher value: a multiple of both subdivisions (Figure 2B). Thus, the value of rhythmic complexity demonstrates the extent and variety of subdivisions within one measure.

A piece of music can theoretically yield any rhythmic complexity value, but most music is made up of subdivisions of 2 or 3 [12]; above a complexity of 6, it becomes less and less likely that an attempt to interpret rhythm in the sample was successful.

### 2.4 Conversion to Milliseconds

To test how accurately our transcriptions matched the samples, we generated onset data for all transcriptions and recordings in milliseconds from the beginning of the recording. Since our transcriptions were in music notation software, we had to convert them first into MIDI, and then into milliseconds.

To convert our transcriptions into MIDI, we exported the MuseScore file as MIDI and manually ensured the beginning of each measure matched the timecode we had recorded. To convert the original recordings into MIDI, we used an energy trigger in the DAW, which uses MIDI to mark every instance a sound recording exceeds a certain decibel limit determined by the user. Using both audio and visual confirmation in the waveform itself, we manually corrected any errors this algorithm had made. With samples from various species as well as randomly calculated MIDI files, this method of detection ensured all three datasets could be processed with the same software, but it was inefficient.

MIDI files are guided by tempo, and the resolution is not milliseconds but pulses per quarter note (PPQN). Using the time, PPQN, and tempo recorded at each note's onset, we converted all note onset data to milliseconds.

With these onset data, we calculated:

transcribednoteonset−recordingnoteonset=offset

Thus, an offset of 0 ms would indicate that a note's onset in the recording perfectly coincided with that of its transcribed version. A transcription in which every note has an offset of 0 ms from the sound it describes in the recording would be exactly accurate; all onset time information from the recording would be preserved in the transcription. However, even in the case that a performer reads from music notation to inform their performance, like the snare pieces, robotic accuracy is impossible; therefore, a nonzero offset can be expected in every measure transcribed. A positive offset indicates that the transcribed note onset occurred later than its counterpart in the recording, and a negative offset indicates that a transcribed note onset occurred earlier than its counterpart in the recording.

### 2.5 Analyses

**FIGURE 3:**
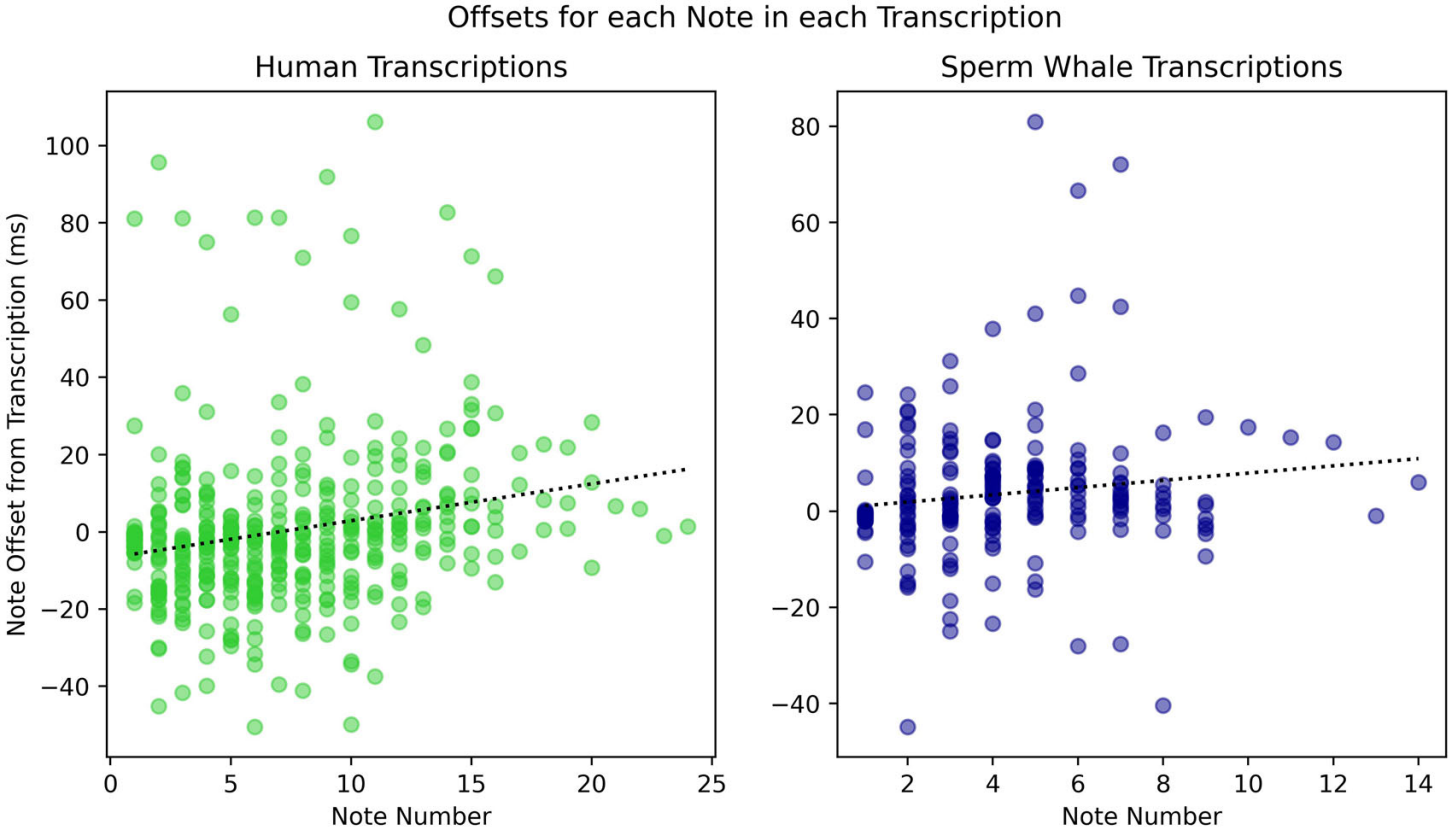
Offset between transcription and recording for all notes superimposed, over the *x*‐axis, each note's number within the measure. 0 means a sound onset occurred at the exact time as a perfect performance of the notated rhythm. A positive value means the onset occurred earlier, and a negative value means the onset in the recording came after the transcribed note. The dotted line shows a linear fit with equal point weighting.

We analyzed the resulting data to provide material for exploring tempo variation and motivic variation. We plotted each dataset's note offset over the order in which the note occurred in its respective measure to create Figure 3. The goal of Figure 3 was to identify broad trends of tempo variation within measures.

We cross‐referenced the phrases of three or more notes that we had flagged, calculating the number of phrase repetitions within each dataset. This also served as a broad starting point in identifying motives.

## 3 Results and Discussion

### 3.1 Statistics

#### 3.1.1 Human

**FIGURE 4:**
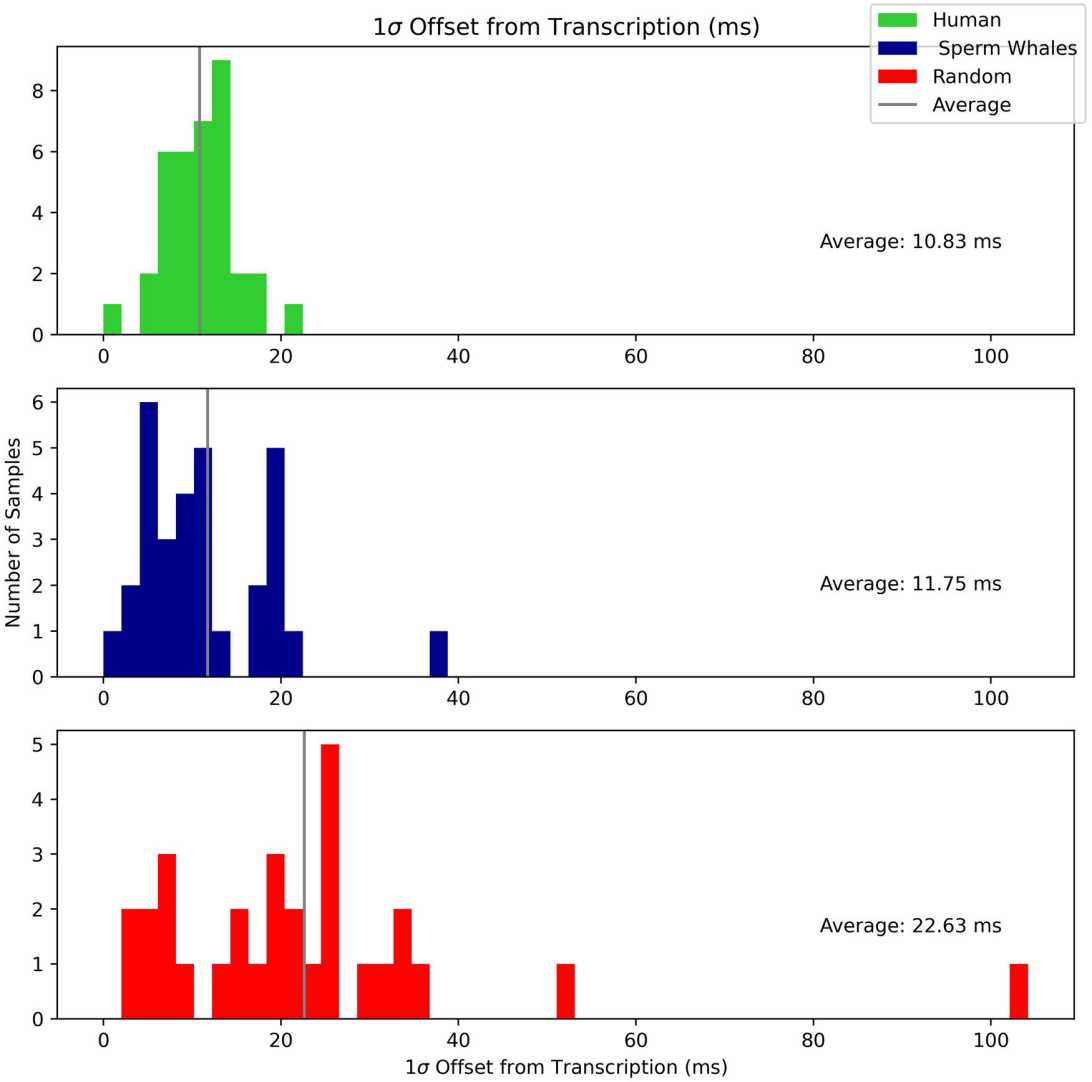
Histogram of the standard deviation per measure of every offset in the measure. Offset: transcribed note onset (ms)—recording note onset (ms). The mean offset is shown as a vertical line, and the numeric value given per group. Median human: 10.99 ms, sperm whale: 10.19 ms, random: 20.51 ms.

The human recordings yielded by far the most frequent onsets, with an average of 12.4 notes per measure transcribed. Humans also deviated the least from our transcriptions, with an average standard deviation of 10.83 ms, as seen in Figure 4. The mean rhythmic complexity was 3.5, and a plurality of measures had a complexity of 3. This means that in our transcribed interpretation, the smallest IOI of many measures had a 3:1 or 3:2 relationship with the largest IOI of that measure. This is in agreement with the observation that most human music is made up of small integer subdivisions of a beat [12].

#### 3.1.2 Sperm Whale

The sperm whale codas we transcribed scored much closer to humans than they did to random rhythms in complexity and offset. A majority of codas had a rhythmic complexity of 1, which indicates isochrony, and the whole dataset averaged a complexity of 1.9. Sperm whales’ standard deviation from our transcriptions was higher than humans by 0.92 ms.

#### 3.1.3 Random

The random rhythms had the highest complexity and the highest offset from our transcriptions. The mean complexity of all random measures was 9.1, meaning that the subdivisions we needed to accurately transcribe the samples were, on average, much higher than the other datasets, and such complex transcriptions were still less accurate than the other datasets. Based on these results, our efforts to interpret rhythm in random sounds were unsuccessful, as expected. As Table 2 shows, random rhythms yielded the fewest and least frequent notes out of the three sources, with an average of 4.9 notes per transcriber‐assigned measure.

**TABLE 2:** Basic statistics of the three datasets.

Group	Total recordings	Total time (m:s)	Recording days	Individuals represented	Total measures	Total notes	Avg. tempo (bpm)	Avg. complexity
Random	3	1:56	N/A	N/A	30	147	103.6	9.1
Sperm whale	5	1:49	5	6	31	207	143.6	1.9
Human	3	1:24	3	3	36	445	139.8	3.5
All	11	5:09			97	799		

*Note*: Average complexity of each group, as explained above and in Figure 2.

### 3.2 Analyses

#### 3.2.1 Overview

Revisiting the question of whether Western music notation can accurately describe sperm whale codas with these data in mind, we can conclude that the process was successful for the small sample size at hand. The transcriptions were similarly accurate to their sources as in the human dataset, and they did not have to be more complicated to achieve this accuracy.

As discussed, the level of accuracy of music notation does not indicate whether all information encoded by such notation is present in the samples transcribed. Time signatures and measures are present in every transcription, but these are the products of human interpretation. Even so, some sources, like the random rhythms, cannot be notated with similar accuracy; therefore, some information can be gleaned from the further success or failure of these methods on a wider scale. Namely, it would indicate the presence of an isochronous pulse—whereas meter describes an isochronous pulse *and* a hierarchy of rhythmic structure [2]—even in heterochronous rhythms, and would thus corroborate other observations of rhythmicality in sperm whale codas [8, 9].

What kinds of analysis that have been applied to Western music notation can we now apply to these notated vocalizations? Given the small sample size, the discussions and statistics from these analyses are meant to demonstrate possibilities rather than offer significant results.

#### 3.2.2 Tempo Variation

In human musical circles, when tempo modulation occurs within a measure—generally unconsciously—it is called “rushing” or “dragging” the beat. Tempo variation occurs independently of changes in note frequency [11]. For example, a 3+1 coda decreases in onset frequency over time, but it still may follow a consistent tempo if all four clicks fall on either a beat or a subdivision of the beat. Tempo variation is also separate from differences in tempo between rhythms; a rhythm at a slow tempo can still rush, and one at a fast tempo can still drag. We will continue to use the terms “rush” and “drag” because they best describe tempo modulation that is local to one measure, but the examples below are not necessarily the results of error in the individuals recorded.

Figure 3 plots each recording's offset throughout a measure from its respective transcription, which represents a mathematically perfect expression of the rhythm we notated. Notably, the human performances in our dataset exhibit a tendency to rush, or increase in tempo, from the beginning of a measure to the end. Our sperm whale dataset also tends to click earlier (relative to a steady tempo) the further into a coda that click lands, although the relationship between a note's position in the measure and its deviation from “perfect” is inconclusive. Our random dataset showed no significant tendency to either rush or drag.

This is a granular way to examine vocalizations that becomes available when tempo is taken in BPM or Hz rather than seconds. Especially if tempo assignment is automated to select the value that minimizes total offset among all clicks in the coda, one can analyze the results in a similar way to identify changes comparable to rushing or dragging in humans.

A change in tempo within one coda may be called intracoda tempo modulation. It is comparative; if every instance of a rhythm type shares a similar offset per position in the measure, then this would simply mean that the transcription describing it is inaccurate. However, if offset per position in the measure varies between individuals, regions, or other factors, such variation can be defined as intracoda tempo modulation.

With tempo data that measure the frequency of a perceived beat, can the tempi of different rhythm types be compared? We attempted this in a case study of one recording.

One of the sperm whale recordings (7200900F) contained two isochronous coda types at different onset frequencies. The metadata described two animals exchanging codas of 9 clicks and 7 clicks, respectively [16]. We differentiated whales based on amplitude and visual examination of the waveform generated—each had a different reflective pattern. Instances of overlap allowed us to confirm the presence of multiple animals. Focusing only on ICI, the codas were different in frequency and number of clicks. However, they had very similar average coda lengths; whale 1's (9 clicks) was 0.50 s, and whale 2's (7 clicks) was 0.52 s. Furthermore, when interpreting different rhythmic subdivisions in the two codas (4 and 3 clicks/beat, respectively), both codas had the same number of beats, and the tempi were much closer, especially by the end of the recording.

Also described in the metadata was a third animal in the background making steady clicks. We had a clear enough sound to record the onset of every click from whale 3, and calculate a tempo in BPM extrapolated from the duration of every ICI. The average tempi of whales 1, 2, and 3 in the recording were 121.4, 114.8, and 111.9 BPM, respectively.

This example brings up the possibility that tempi of different rhythm types can be compared, as well as the possibility that the tempi of codas can be compared with the tempi of other sound sources in the ocean. Questions regarding how coda tempo changes in response to external sources of rhythm could potentially be tested in this way.

The problem is that the interpretation of these sources cannot be standardized in the same way that transcriptions of the same coda type can—thus, the subjectivity of such interpretations is not mitigated in this case. The comparison of different rhythm types’ tempi cannot be seriously attempted, then, unless beat interpretations of all sources involved are tested, accepted, and standardized.

#### 3.2.3 Motivic Variation

A motive in Western music theory is a short series of notes that undergoes motivic variation, a process by which the motive is reiterated, often in altered states, to generate new sequences [22]. Motivic patterns are found in a majority of human music [12]. The repetition of coda rhythm types is partly analogous to the repetition of motivic patterns [23], but there are fewer observations of cetacean communication that are analogous to the alteration of a motive [7, 24].

To gauge the potential presence of motivic patterns in our transcriptions, we examined the phrases of three or more notes that we had previously marked as repeating somewhere else in that species’ dataset. Identifying a rhythm requires three or more events [25, 26], and these three‐note phrases acted as a very broad net for potential motives.

Nine and a half percent of notes in the random rhythm transcriptions belonged to a phrase that repeated elsewhere in the dataset. The proportion of notes in repeated phrases for sperm whales was 82.1%, and for humans, 88.8%. In the sperm whale samples, repetition of coda rhythm types accounted for all of the recurring phrases, whereas in the human rhythms, smaller phrases within measures were repeated more often than entire measures were. Therefore, using this method, the only direct repetition we found in sperm whale codas was the repetition of rhythm types themselves.

**FIGURE 5:**
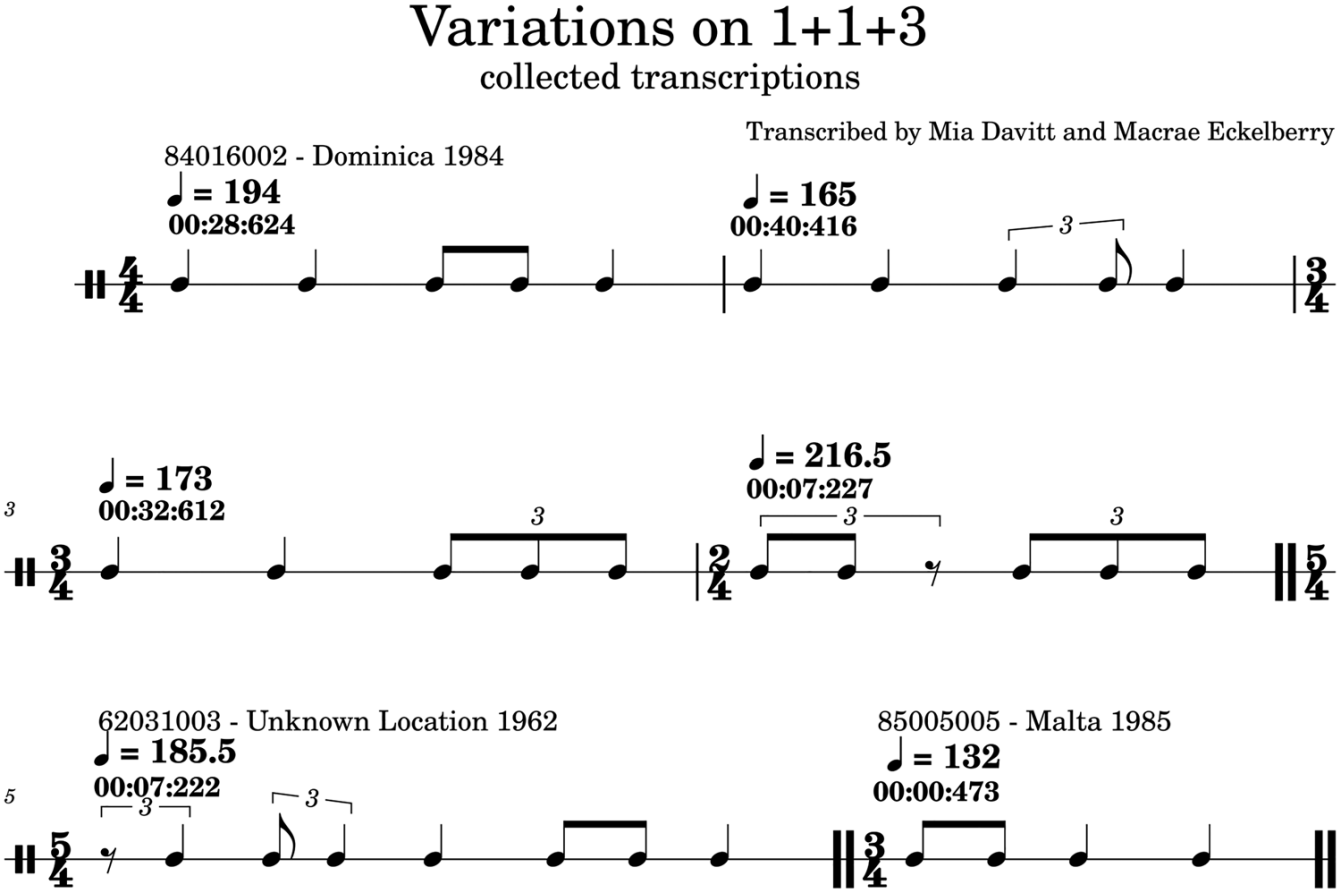
A collection of measures from three transcribed audio recordings of sperm whale codas. The text above each measure shows the reference number and location of recording, as well as tempo, and time code of the first note in the coda.

Next, we used a more hands‐on approach in analyzing selected codas through the lens of motivic variation. Figure 5 is a collection of six codas across three recordings.

Measures (m.) 1–4, all from the same recording, bear a structural similarity to the coda rhythm type 1+1+3 [27], but their transcriptions vary. Measures 1 and 2 are rhythmically identical except for the second‐to‐last note, which is delayed in m. 2 compared to m. 1. Measure 3 shares similarities with both m. 1 and m. 4. In fact, the last three notes of m. 1, 3, and 4 are all isochronous within the respective measures. They differ only in how the final three notes relate to the tempo established by the first two.

The comparisons made above do not necessarily require music notation. Measures 1 and 3 can be compared via various methods: categorical rhythm (m. 1−1:2, and m. 3−1:3), or compression/expansion (m. 3's last three clicks compressed compared to m. 1) [8, 14]. Music notation, in this case, acts instead as an aid for easy comparison—similarities that might not otherwise be found can be identified, then tested using other means.

Measures 5 and 6 are both different rhythm types than 1+1+3, due to the order and proportion of ICIs in each, but still they can be compared to previous measures using common concepts in motivic variation. To focus on m. 5, for example: temporarily ignoring the first and third note, the rhythm of every other note exactly matches m. 1. The presence of the first and third note makes it a different rhythm type, but the similarity remains.

The rhythmic values of m. 6 match the first four note values of m. 1 in retrograde order: the first note of m. 1 corresponds with the last note of m. 6, the second with the third, and so on.

Motivic variation is an imperfect tool in application to these data for two reasons. First, it implies a hierarchy in which one sequence is defined as the original motive and the rest are alterations, where there is little evidence to suggest that the same hierarchy exists between sperm whale rhythms. Second, it is designed to analyze composed works, and it assigns intention to its findings, which is problematic when discussing animal vocalizations.

However, the first aspect of motivic variation discussed, pattern repetition, has been widely studied in sperm whales and has even been compared to musical motives [23]. Separately, variation among different instances of the same coda rhythm types has been observed [7, 14, 28]. A mode of analysis that adapts the kinds of changes studied in motivic variation—without assuming an origin—could provide a new way to find connections between coda types, as well as variations within coda types.

### 3.3 Limitations

Our methods of transcription yielded a tendency for notes that we interpreted as coinciding with the beat to have a much lower offset than notes we interpreted as syncopated. The former were the notes that, when transcribing the measure, we used to make sure the click of the metronome lined up with the rhythm of the recording. The latter were notated as subdivisions of the beat, and we did not align them directly with the click of a metronome. We retranscribed one recording of sperm whale codas, deliberately choosing a different tempo and beat that would coincide with sounds we had previously interpreted as syncopated. Similarly to the changeable beat perception of polyrhythms, this transcription held the same informational content but with a different perception of beat. The alternate transcription showed the same tendency for notes that lined up with the new beat to be more accurate, even though the set of notes that met that criterion was different. Because of this, we can surmise that the correlation between syncopation and offset is most likely caused by limitations in our methods, rather than a real tendency in the recording.

### 3.4 Note on Random Rhythm

Another point to note is that the average onsets per second we chose for random rhythms was not comparable to the onset frequency of codas. Since our methods of analysis focused on measures rather than interonset frequency, the criteria on which we compared the three datasets were not altered by this difference.

### 3.5 Suggestions for Future Work

The methods used in this study were cumbersome and in need of streamlining. This was due to the tools being used and the nature of the data collected.

Regarding the tools: MuseScore 4 is an open source compositional and arranging tool—a custom program solely for the purpose of transcribing codas would greatly streamline the process. Once a sufficient sampling of one coda type has been transcribed, the process can be automated for all other instances of that type in a dataset, marking the most inaccurate transcriptions for further scrutiny.

The sperm whale data we accessed consisted of raw audio recordings, and our methods needed to analyze randomly generated rhythms, human drumming, and sperm whale codas using the same software. Studies focusing on one species instead of multiple could use much faster methods of onset detection that would circumvent the need for MIDI conversion. Alternatively, there are large datasets available with click onset times recorded, location and coda type, and more data that would make further transcriptions much simpler and the resulting analyses more robust.

Many of the analyses discussed require an isochronous beat to be perceived in vocalizations—but beyond that, they do not need to be transcribed in music notation. The first step, assigning an isochronous beat to nonhuman rhythm, can be used for different applications better suited to different needs.

Also important is an understanding of how rhythmic phenomena appear when using different methodologies. A categorical rhythm study of sperm whale codas has observed ICI relationships at near‐integer ratios in some coda types, slightly offset from the actual integer ratio [8]. Would an instance of intracoda tempo modulation yield a similarly offset ICI ratio? Comparative tests of different methods will help to explain and corroborate observations found in other frameworks.

Further research on sperm whale vocalization may apply musical transcription and analysis tools to a larger selection, and examine variables that were not explored here, such as amplitude, timbre, or differences in expression between clans.

## 4 Conclusions

For this case study, we used a human musical language to describe three datasets—sperm whale, human, and random. We employed subjective methods of interpreting rhythm, but we took care to maintain consistency and to test the accuracy of the resulting transcriptions. The standard deviation offset between transcription and audio in the sperm whale dataset was much closer to that of the human dataset than the random rhythms. The complexity of rhythmic subdivision was lowest in sperm whales, due in part to a higher presence of isochronous rhythms in our samples.

Although Western music notation necessarily communicates a metric hierarchy, it has various potential use cases in animal communication, such as facilitating granular measurements in tempo and motivic analysis. The analytical concepts presented here—tempo variation and motivic variation—are laid out as possibilities for further exploration. With a system that automates the transcription process based on the existing classifications of rhythm type, for example, one would greatly increase the scale of data available and would also pinpoint the codas that are the least well described by that classification.

Our research provides a methodology that, applied to a larger sample size, can corroborate or challenge observations of rhythmicality in sperm whale vocalizations, and it offers a new framework with which to view animal communication.

## Author Contributions

**Mia Davitt**: Conceptualization, data curation, formal analysis, investigation, methodology, validation, project administration, visualization, writing – original draft, writing – review and editing. **Macrae Eckelberry**: Conceptualization, data curation, investigation, methodology. **Max Davitt**: Software. **Lara S. Burchardt**: Visualization, funding acquisition, supervision, writing – review and editing.

## Conflicts of Interest

The authors declare no competing interests.
